# Pathological Spectrum of Melanoma: A Nine-Year Retrospective Analysis of Tumor Characteristics Across Diverse Anatomic Sites

**DOI:** 10.7759/cureus.75873

**Published:** 2024-12-17

**Authors:** Indhu Kannan, Rajeswari Kathiah, Karthikeyan V S, Raja A.M

**Affiliations:** 1 Pathology, Meenakshi Mission Hospital and Research Centre, Madurai, IND; 2 Pathology, All India Institute of Medical Sciences, Madurai, Madurai, IND; 3 Radiology, Aarthi Scans and Labs, Madurai, IND; 4 Ophthalmology, All India Institute of Medical Sciences, Madurai, Madurai, IND

**Keywords:** anatomic diversity, lymphovascular invasion, malignant melanoma, melanoma, non-cutaneous melanoma, pathology, retrospective analysis, tumor characteristics, tumour characteristics

## Abstract

Melanoma is a highly aggressive malignancy originating from melanocytes, characterized by its potential to arise in various anatomic locations, both common and rare. The incidence of melanoma has been steadily increasing globally, with variations in clinical presentation, tumor behavior, and prognosis depending on the anatomical site involved. Understanding the diverse pathological spectrum of melanoma is critical for optimizing diagnostic and therapeutic strategies.

This retrospective study provides a comprehensive analysis of melanoma tumor characteristics in 25 patients diagnosed and treated over a nine-year period at Meenakshi Mission Hospital. The study spans a wide spectrum of anatomical sites, including both typical cutaneous locations, such as the thigh and arm, and less common locations, such as the rectum, anal canal, esophagus, and glans penis.

The patient cohort, predominantly male (92%, n=23), had a median age of 64 years (range 32-81 years). Tumor sizes varied from 1.2 cm to 5.1 cm, and ulceration was observed in all cases. Histologically, malignant melanoma was the most frequent diagnosis, with subtypes such as balloon cell melanoma, spindle cell melanoma, and superficial spreading melanoma also represented. Mitotic rates ranged from 5 to 22 mitoses per 10 high-power fields (HPF), reflecting varying levels of tumor aggressiveness. Lymphovascular invasion was noted in 12% of cases (n=3), highlighting the metastatic potential in some patients.

This study underscores the pathological diversity of melanoma, highlighting the importance of thorough diagnostic evaluation and tailored therapeutic approaches. The findings contribute to a better understanding of melanoma's clinical and tumor characteristics across both typical and atypical anatomical sites, with implications for improving diagnostic accuracy and patient management.

## Introduction

Melanoma is a malignant tumor of melanocytes that predominantly occurs in the skin but can also manifest in various non-cutaneous sites. While cutaneous melanoma is well-recognized and studied, cases involving atypical anatomical locations such as the rectum, anal canal, esophagus, and glans penis remain less well understood [1,2]. These melanomas present significant diagnostic and therapeutic challenges due to their rarity and the complex clinical presentations associated with these sites. Additionally, the heterogeneity of tumor characteristics, including ulceration, mitotic rates, and the presence or absence of lymphovascular invasion, further complicates the treatment and prognosis of these patients [3,4].

This study aims to provide a comprehensive retrospective analysis of melanoma cases diagnosed over a nine-year period at a tertiary care center, with a focus on tumor characteristics across both typical cutaneous and atypical non-cutaneous anatomical sites. By analyzing key factors such as tumor size, ulceration, histological subtypes, and mitotic activity, we seek to contribute valuable insights into the pathological diversity of melanoma. This data may inform clinical practice and facilitate a deeper understanding of melanoma’s behavior across a spectrum of locations.

The study’s findings underscore the need for heightened awareness and early diagnostic approaches, particularly for melanomas presenting in less common anatomical sites, where clinical suspicion may be lower. A thorough understanding of the pathological spectrum of melanoma is essential for improving patient outcomes, particularly in settings where non-cutaneous melanomas are encountered.

## Materials and methods

Study setting and design

This retrospective study was conducted at Meenakshi Mission Hospital, a tertiary care center in Madurai, India, over a nine-year period from March 2016 to August 2024. Data collection involved a systematic review of clinical and pathological records of patients diagnosed with melanoma.

Study population

A total of 25 patients with confirmed histopathological diagnoses of melanoma, from both cutaneous and non-cutaneous sites, were included in the study. Patients of any age or gender were eligible. The exclusion criteria involved patients with incomplete medical records or without histopathological confirmation.

Inclusion criteria

Patients of any age and gender with a confirmed histopathological diagnosis of melanoma; Melanoma cases from both cutaneous and non-cutaneous sites, regardless of stage at diagnosis; Patients with complete medical records detailing clinical presentation, tumor characteristics, and histopathological findings; Patients who provided informed consent for inclusion in the study.

Ethical considerations

Ethical approval was obtained from the Institutional Ethics Committee (IEC) of Meenakshi Mission Hospital, Madurai. All patients gave informed consent, and the study adhered to the principles of the Declaration of Helsinki. Patient anonymity was ensured by assigning unique identifiers and anonymizing all data.

Data collection

Data were retrieved from electronic medical records and histopathology archives, including: Demographics: Patient age, sex, and family history of melanoma or skin cancer; Clinical Information: Date of diagnosis, presenting symptoms, and anatomical site of melanoma; Tumor Characteristics: Tumor size, ulceration status, histological subtype, Breslow depth, mitotic rate, and lymphovascular invasion; Histopathological Analysis: Conducted using standard diagnostic criteria with immunohistochemical techniques for confirmation when required.

Statistical analysis

Data were analyzed using SPSS software, version 25. Descriptive statistics summarized demographic and tumor characteristics. Categorical variables were presented as frequencies and percentages, while continuous variables were expressed as means or medians with interquartile ranges. Comparative analyses were performed to assess differences between cutaneous and non-cutaneous melanomas regarding tumor size, mitotic activity, and lymphovascular invasion.

## Results

A total of 25 melanoma cases were analyzed over a nine-year period at Meenakshi Mission Hospital. The patient population comprised mostly males (92%, n=23) and a minority of females (8%, n=2), with a median age of 64 years (range: 32-81 years). Table 1 displays the patient demographics, clinical information, and tumor characteristics in malignant melanoma of the first 13 patients in the study.

**Table 1:** Patient Demographics, Clinical Information, and Tumor Characteristics in Malignant Melanoma of the First 13 Patients in the Study.

Category	Data Points	1.	2.	3.	4.	5.	6.	7.	8.	9.	10	11	12	13
Patient details	Age	64	53	43	41	70	82	55	32	66	70	77	67	55
	Sex	M	M	M	M	M	M	M	M	F	M	M	M	M
Clinical Information	Date of Diagnosis	3-4-2016	06-06-2016	22-08-2016	05-11-2016	16-03-2017	25-04-2017	26-10-2017	20-02-2018	06-07-2018	15-10-2018	12-12-2018	17-05-2019	17-11-2019
	Anatomic Site of Melanoma	Rectum	Rectum	Rectum	Anal canal	Thigh	Rectum	Rectum	Rectum	Vagina	Glans penis	Anal canal	Oral cavity	Scalp
	Symptoms at Presentation	Bleeding per rectum	Bleeding per rectum	Bleeding per rectum	Bleeding per rectum	Blackish discoloration of skin with ulcer	Bleeding per rectum	Bleeding per rectum	Bleeding per rectum	Post-coital bleeding	Blackish discoloration of skin with ulcer	Bleeding per rectum	Blackish discoloration with ulceration	Blackish discoloration of skin with ulcer
	Family History of Melanoma/Skin Cancer	None	None	None	None	None	None	None	None	None	None	None	None	None
Tumor Characteristics	Tumor Size (Maximum Diameter in mm)	4.3 cm	1.2 cm	2.5 cm	1.8 cm	1.8 cm	2 cm	2.4 cm	4 cm	3.4 cm	2.8 cm	3.2 cm	1.9 cm	2 cm
	Ulceration (Y/N)	Y	Y	Y	Y	Y	Y	Y	Y	Y	Y	Y	Y	Y
	Histologic Type	Malignant melanoma	Malignant melanoma	Malignant melanoma	Balloon cell melanoma	Spindle cell melanoma	Malignant melanoma	Malignant melanoma	Malignant melanoma	Malignant melanoma	Malignant melanoma	Malignant melanoma	Malignant melanoma	Malignant melanoma
	Breslow Depth (in mm)													T2
	Mitotic Rate (Per mm²)	7/10HPF	5/10HPF	8/10HPF	12/10HPF	5/10HPF	11/10HPF	7/10HPF	6/10HPF	8/10HPF	6/10HPF	10/10HPF	7/10HPF	8/10HPF
	Lymphovascular Invasion (Y/N)	N	N	N	N	Y	N	N	N	N	N	N	Y	N

Table 2 displays the patient demographics, clinical information, and tumor characteristics in malignant melanoma of the last 12 patients in the study.

**Table 2:** Patient Demographics, Clinical Information, and Tumor Characteristics in Malignant Melanoma of the Last 12 Patients in the Study.

Category	Data Points	14.	15.	16.	17.	18.	19.	20.	21.	22.	23.	24.	25.
Patient details	Age	65	56	45	66	71	76	62	72	64	56	81	72
	Sex	M	M	M	F	M	M	M	M	M	M	M	M
Clinical Information	Date of Diagnosis	05-06-2021	17-08-2022	23-10-2022	04-03-2023	14-07-2023	17-08-2023	22-12-2023	05-03-2024	19-05-2024	22-05-2024	06-06-2024	16-08-2024
	Anatomic Site of Melanoma	Rectum	Rectum	Rectum	Esophagus	Nasal cavity	Anal canal	Arm	Glans penis	Rectum	Anal canal	Anal canal	Rectum and anal canal
	Symptoms at Presentation	Bleeding per rectum	Bleeding per rectum	Bleeding per rectum	Dysphagia	Nasal mass	Bleeding per rectum	Blackish discoloration with ulceration	Blackish discoloration of skin with ulcer	Bleeding per rectum	Bleeding per rectum	Bleeding per rectum	Bleeding per rectum
	Family History of Melanoma/Skin Cancer	None	None	None	None	None	None	None	None	None	None	None	None
Tumor Characteristics	Tumor Size (Maximum Diameter in mm)	4.1 cm	4.2 cm	3.3 cm	4.3 cm	2.3 cm	5.1 cm	3.3 cm	2.1 cm	3.4 cm	2.1 cm	2.2 cm	3 cm
	Ulceration (Y/N)	Y	Y	Y	Y	Y	Y	Y	Y	Y	Y	Y	Y
	Histologic Type	Malignant melanoma	Malignant melanoma	Malignant melanoma	Malignant melanoma	Malignant melanoma	Malignant melanoma	Superficial spreading melanoma	Malignant melanoma	Malignant melanoma	Malignant melanoma	Malignant melanoma	Malignant melanoma
	Breslow Depth (in mm)								T2				
	Mitotic Rate (Per mm²)	12/10HPF	15/10HPF	11/10HPF	22/10HPF	16/10HPF	17/10HPF	7/10HPF	8/10HPF	10/10HPF	13/10HPF	16/10HPF	7/10HPF
	Lymphovascular Invasion (Y/N)	N	N	N	Y	N	N	N	N	N	N	N	Y

Anatomic sites

The melanomas presented in a variety of anatomical sites: Rectum: nine cases (36%), anal canal: five cases (20%), other non-cutaneous sites: esophagus, nasal cavity, oral cavity, and glans penis (16% collectively). Cutaneous sites: Thigh, arm, and scalp (12% collectively)

Symptoms at presentation

The most common presenting symptom was bleeding per rectum, reported in 11 cases (44%). Other symptoms included blackish discoloration with ulceration, post-coital bleeding, dysphagia, and nasal mass, depending on the site of the melanoma.

Tumor Size

Tumor sizes varied from 1.2 cm to 5.1 cm in maximum diameter.

Ulceration

All 25 cases (100%) presented with ulceration.

Histologic Types

**Figure 1:**
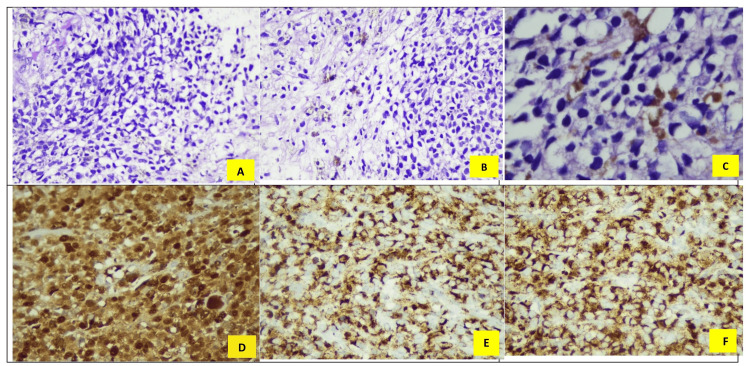
Histopathology of Malignant Melanoma (H&E stain, 100× magnification). A) Image shows atypical melanocytes with nuclear pleomorphism, hyperchromasia, and prominent nucleoli; B) Histopathology of malignant melanoma (H&E stain, 100× magnification), the image shows atypical melanocytes with nuclear pleomorphism and prominent nucleoli. Tumor cells are arranged in nests with scattered brown melanin pigment, typical of malignant melanoma; C) Histopathology of Malignant Melanoma (H&E stain, 400× magnification). This high-power image highlights atypical melanocytes with nuclear pleomorphism and prominent nucleoli. Scattered melanin pigment is evident within the tumor cells, consistent with malignant melanoma; D) Immunohistochemistry of Malignant Melanoma (S-100, 400× magnification), E) Immunohistochemistry of Malignant Melanoma (HMB-45, 400× magnification), F) Immunohistochemistry of Malignant Melanoma (Melan-A, 400× magnification)

The predominant histologic type was malignant melanoma (88%, n=22) (Figures 1A-1C).

**Figure 2:**
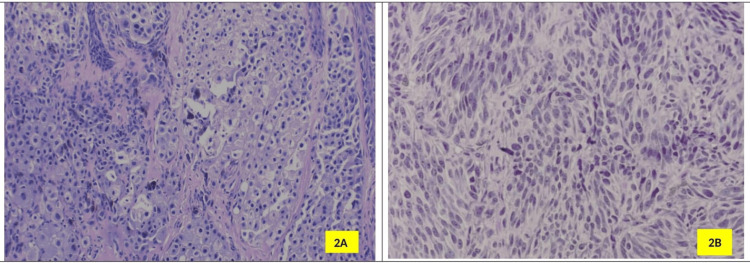
Histopathology of Rare Histologic Types of Melanomas. A) Histopathology of balloon cell nevus showing nests of ballooned melanocytes with pale, vacuolated cytoplasm (H&E stain, 400× magnification); B) Histopathology of spindle cell nevus showing fascicles of spindle-shaped melanocytes with elongated nuclei and scant cytoplasm (H&E stain, 400× magnification).

Rare subtypes such as balloon cell melanoma, spindle cell melanoma, and superficial spreading melanoma accounted for the remaining cases (Figures 2A-2B).

Immunohistochemistry

The cases were positive for S100 (Figure 1D), HMB 45 (Figure 1E), and Melan A (Figure 1F).

Mitotic Rates

Mitotic activity varied significantly among the cases, ranging from 5 to 22 mitoses per 10 high-power fields (HPF).

Lymphovascular Invasion

Present in 12% of cases (n=3).

This study highlights the wide anatomical diversity of melanoma presentation, with a predominance in non-cutaneous sites like the rectum and anal canal. Despite this diversity, most tumors displayed aggressive histopathological features, including ulceration and high mitotic activity.

## Discussion

The findings from this nine-year retrospective study underscore the wide anatomical diversity of melanoma and its pathological characteristics across both typical and atypical sites. Melanoma is typically considered a cutaneous malignancy, with most studies focusing on its presentation in sun-exposed areas like the skin of the limbs, back, and face [5]. However, this study demonstrates the significant occurrence of melanoma in non-cutaneous sites, such as the rectum, anal canal, esophagus, and glans penis, which pose unique diagnostic and therapeutic challenges due to their rarity and often delayed presentation [6,7].

One of the most notable findings of this study is the predominance of non-cutaneous melanomas, which accounted for over 70% of the cases analyzed. The rectum and anal canal were the most commonly affected non-cutaneous sites. These cases often presented with symptoms such as rectal bleeding, which can resemble other gastrointestinal conditions, resulting in diagnostic delays. This observation is consistent with other studies, such as those by Adinarayan et al. and Panda et al., which also report similar findings [8,9]. The relatively high proportion of male patients (92%) in this cohort highlights the potential influence of gender-related factors in the development of melanoma, particularly in non-cutaneous locations. Research by Premi et al. and others has reported similar trends [10], though additional studies with larger sample sizes are needed to investigate this further.

Tumor characteristics in this study revealed aggressive features in nearly all cases, with ulceration observed in 100% of the tumors and high mitotic activity (ranging from 5 to 22 mitoses per 10 HPF) across the cohort. Ulceration and mitotic rate are well-established prognostic factors in melanoma, correlating with a higher risk of metastasis and poorer outcomes [11,12]. The high frequency of these aggressive features highlights the need for vigilant monitoring and early intervention, even in atypical presentations of melanoma [13].

Lymphovascular invasion, although observed in only 12% of cases, further emphasizes the metastatic potential of melanoma in non-cutaneous sites. Previous studies have shown that lymphovascular invasion is a critical step in the metastatic process, leading to the spread of melanoma to regional lymph nodes and distant organs. This is particularly concerning for patients with non-cutaneous melanomas, who may not receive adequate screening or diagnostic workups due to the rarity of their presentations. For instance, anorectal melanomas are known to have a higher propensity for distant metastasis, often leading to poor prognoses [14].

Histologically, malignant melanoma was the most frequent diagnosis in this study, with rare subtypes such as balloon cell melanoma, spindle cell melanoma, and superficial spreading melanoma also represented. These histological variants have been noted to have varying prognostic implications. For instance, balloon cell melanoma is often associated with a more indolent course, while spindle cell melanoma tends to be more aggressive [15]. The presence of such subtypes highlights the histopathological diversity of melanoma, underscoring the importance of accurate histological diagnosis to guide appropriate management.

The findings of this study underscore the critical need for heightened awareness and clinical suspicion of melanoma, especially in non-cutaneous sites where it may present with atypical symptoms. Clinicians must consider melanoma in the differential diagnosis of pigmented lesions or ulcerations in unusual anatomical locations, such as the gastrointestinal or genitourinary tract. Given the aggressive nature of melanoma observed in this cohort, early detection and timely intervention remain paramount in improving patient outcomes.

This study also has important implications for public health and preventive medicine. While cutaneous melanoma is closely associated with sun exposure, the etiology of non-cutaneous melanomas is less clear, with potential links to genetic factors, chronic inflammation, and environmental exposures [16]. Future research should focus on elucidating these risk factors to better inform screening and prevention efforts, particularly for populations at higher risk for non-cutaneous melanoma.

Limitations

There are several limitations to this study that warrant consideration. First, the small sample size limits the generalizability of the findings, particularly regarding the less common non-cutaneous melanoma subtypes. Additionally, the retrospective nature of the study may introduce biases related to incomplete data collection or inconsistent reporting in medical records. Finally, the study was conducted at a single tertiary care center, which may not reflect the broader population trends seen in different geographic regions or healthcare settings.

## Conclusions

In conclusion, this study highlights the pathological spectrum of melanoma across both typical and atypical anatomical sites, with a particular focus on non-cutaneous presentations. The high frequency of aggressive features, such as ulceration, high mitotic rates, and lymphovascular invasion, emphasizes the need for early recognition and intervention in these patients. Increased awareness among clinicians, coupled with improved diagnostic tools, will be key to enhancing outcomes for patients with melanoma, particularly those with non-cutaneous involvement. Further research is needed to better understand the risk factors and optimal management strategies for these rare but clinically significant melanomas.

## References

[1] Williams PF, Olsen CM, Hayward NK, Whiteman DC (2011). Melanocortin 1 receptor and risk of cutaneous melanoma: A meta-analysis and estimates of population burden. Int J Cancer.

[2] Seiberg M (2001). Keratinocyte-melanocyte interactions during melanosome transfer. Pigment Cell Res.

[3] Morgan AM, Lo J, Fisher DE (2013). How does pheomelanin synthesis contribute to melanomagenesis?: Two distinct mechanisms could explain the carcinogenicity of pheomelanin synthesis. Bioessays.

[4] Mitra D, Luo X, Morgan A (2012). An ultraviolet-radiation-independent pathway to melanoma carcinogenesis in the red hair/fair skin background. Nature.

[5] Premi S, Wallisch S, Mano CM (2015). Chemiexcitation of melanin derivatives induces DNA photoproducts long after UV exposure. Science.

[6] Read J, Wadt KA, Hayward NK (2016). Melanoma genetics. J Med Genet.

[7] Robles-Espinoza CD, Roberts ND, Chen S (2016). Germline MC1R status influences somatic mutation burden in melanoma. Nat Commun.

[8] Adinarayan M, Krishnamurthy SP (2011). Clinicopathological evaluation of nonmelanoma skin cancer. Indian J Dermatol.

[9] Panda S (2010). Nonmelanoma skin cancer in India: Current scenario. Indian J Dermatol.

[10] Mohania D, Chandel Chandel, Kumar P (2017). Ultraviolet radiations: Skin defense-damage mechanism. Adv Exp Med Biol.

[11] Shain AH, Bastian BC (2016). From melanocytes to melanomas. Nat Rev Cancer.

[12] Rebecca VW, Sondak VK, Smalley KS (2013). A brief history of melanoma: From mummies to mutations. Melanoma Res.

[13] Lee C, Collichio F, Ollila D, Moschos S (2013). Historical review of melanoma treatment and outcomes. Clin Dermatol.

[14] Scolyer RA, Long GV, Thompson JF (2011). Evolving concepts in melanoma classification and their relevance to multidisciplinary melanoma patient care. Mol Oncol.

[15] Mehnert JM, Kluger HM (2012). Driver mutations in melanoma: Lessons learned from bench-to-bedside studies. Curr Oncol Rep.

[16] Li C, Hu Z, Liu Z (2006). Polymorphisms in the DNA repair genes XPC, XPD, and XPG and risk of cutaneous melanoma: A case-control analysis. Cancer Epidemiol Biomarkers Prev.

